# The relationship between major life events and non-suicidal self-injury among college students: the effect of rumination and body image

**DOI:** 10.3389/fpubh.2023.1308186

**Published:** 2024-01-17

**Authors:** Qian Qin, Guangni Yang, Yue Li, Wanchun Wu, Jianping Wang, Ziyao Chen, Xiaohua Kong, Wei Zhang, Hongyu Zou

**Affiliations:** ^1^Center for Studies of Psychological Application, School of Psychology, South China Normal University, Guangzhou, China; ^2^School of Psychology, Shenzhen University, Shenzhen, China; ^3^Department of Psychology, Lingnan University, Hong Kong, China; ^4^College of Teacher Education, South China Normal University, Guangzhou, China; ^5^Journal of South China Normal University, South China Normal University, Guangzhou, China

**Keywords:** major life events, NSSI, rumination, body image, college students

## Abstract

**Background:**

Non-suicidal self-injury (NSSI) poses a growing risk to public health worldwide. While numerous studies have identified major life events as key risk factors for NSSI, the mechanisms by which emotional and cognitive problems mediate or moderate this relationship remain unclear. To enhance the understanding of this field, we will draw upon the cascade theory of self-injury and the benefits and barriers model, to examine the relationship between major life events and NSSI, as well as the effect of rumination and body image.

**Methods:**

A sample of 2,717 college students (M_age_ = 19.81 years; SD = 1.09) participated in this study and anonymously completed the questionnaires. The moderated mediation model were conducted using Model 4 and Model 15 of the Process macro program in SPSS.

**Results:**

The results showed that rumination mediated the positive relationship between major life events and NSSI. Furthermore, body image was found to moderate both the relationship between major life events and NSSI, as well as the relationship between rumination and NSSI.

**Conclusion:**

The current findings suggest that rumination is an important mediator in the relationship between major life events and NSSI among college students. Teachers, parents, and researchers should recognize the important role of body image self-perceptions of college students and actively promote a healthy and accurate body image.

## 1 Introduction

Non-suicidal self-injury (NSSI) is frequently acknowledged to be common in adolescence (10–24 years old) (1, 2). In recent years, NSSI among adolescents has emerged as a major global public health concern (3). Non-suicidal self-injury refers to an intentional and self-injurious behavior that is not accompanied by suicide ideation and is not socially acceptable. The common expressions of NSSI encompass self-inflicted actions such as cutting, burning, and scratching (4). The mentioned behavior often occurs in adolescents and young adults and has the potential to cause serious harm to their physical and mental well-being (5). Research studies showed that 17.7% of college students have engaged in NSSI on at least one occasion throughout their lifetime (6). Meanwhile, in China, a nationally representative sample of over 150,000 adolescents noted that 13.4% of the participants had engaged in NSSI at least three or more times within a single year (7). Non-suicidal self-injury is an important risk factor for adolescent suicide, with one study noting that adolescents who engaged in NSSI were associated with an increase of 17 times in suicide behavior than their counterparts who did not engage in NSSI (8). Hence, it is crucial to improve understanding of the causes and potential mechanisms underlying NSSI in order to promote the mental well-being of adolescents and college students.

Major life events refer to a range of significant events that occurred in the lives of adolescents within the preceding year, potentially having a substantial impact on them (9). These events cover a variety of experiences, including traumatic experiences, loss, and major transitions (10). Research showed that major life events have been identified as important risk factors for NSSI. When individuals are exposed to major life events, they will develop more negative emotions and use poor coping styles when the emotions are not resolved; negative life events are the trigger for negative emotions and risky behaviors in individuals (11). At the same time, the experiential avoidance theory posits that individuals who experience negative emotions when confronted with negative events are especially susceptible to engaging in NSSI. To avoid these negative emotions, individuals often choose to engage in NSSI as a way of attaining immediate emotional relief and avoiding the unpleasant experience (12). Additionally, a longitudinal study consisting of three waves of data indicated that major life events at T1 were found to have a direct impact on the occurrence of NSSI at T3 (13). In recent years, numerous studies have explored the effects of NSSI on various psychological factors, such as depression (14), anxiety (15), and cyberbullying (16). These studies have contributed to our understanding of NSSI. Nevertheless, the specific mediating and moderating mechanisms of this phenomenon remain unclear and need to be further investigated.

Rumination, a maladaptive cognitive process characterized by the repetitive and intrusive thought of negative experiences (17), was thought to be a key cognitive process in maintaining NSSI (18). The emotional cascade theory about NSSI pointed out that when an individual is exposed to an adverse event, they are prone to the presence of high arousal negative emotions. This high arousal negative emotion will continue to fester in the human brain and cannot be released, thus allowing rumination to occur, and when rumination reaches a certain level and the individual is unable to cope with the psychological pain, they will relieve themselves of the psychological pain with the immediately available NSSI (physiological pain), thereby increasing the likelihood of engaging in NSSI (19). According to emotion appraisal theory, emotions arise from interpretations and explanations of the environment of people in which they find themselves. According to Richard Lazarus, there are two main types of reactions to major life events: automatic, unconscious, and rapidly activated emotional responses, and conscious emotional responses related to how to cope, such as rumination (20). A longitudinal study that included 1,065 samples noted that major life events can predict rumination, while rumination played an important mediating role in the relationship between major life events and mood problems (depression and anxiety) (21). Numerous studies have indicated a significant positive relationship between rumination and NSSI among college students (22, 23). Furthermore, a meta-analysis included 46 studies additionally demonstrated that rumination exerted a significant influence on NSSI (24). Although numerous studies have pointed out that emotions mediate the relationship between major life events and NSSI (25). There is a limited body of research that examines the role of rumination, which encompasses negative emotions and cognitive functioning, in the relationship between major life events and NSSI. Thus, we hypothesize that rumination mediated the relationship between major life events and NSSI in college students.

In addition, the benefits and barriers model of NSSI pointed out that Body Perceptions for Identity are a significant barrier to NSSI and serve as an important protective role (26). Body image refers to an individual’s subjective perception of the esthetic or sexual appeal of their own body. It places greater emphasis on self-perception of individuals rather than conforming to social standards (27) and serves as a fundamental element of self-identity (28). Individuals with high body intention toward their physical appearance are more likely to develop a strong attachment to their bodies while also being less likely to engage in NSSI (29). This theory suggests that having a positive body image may be an important protective factor in the development of NSSI problems. This protective function is believed to moderate the effects of adverse experiences and negative emotions on NSSI. Research has been indicated that body image can moderate the relationship between emotional maladjustment and NSSI. However, there is a lack of research to validate whether body intention moderates the relationship between major life events and NSSI, as well as the relationship between rumination and NSSI (30). We therefore hypothesize that body image can moderate the relationship between major life events and NSSI, as well as the relationship between rumination and NSSI. However, the specifics of the moderating effect need to be further validated.

**Figure 1:**
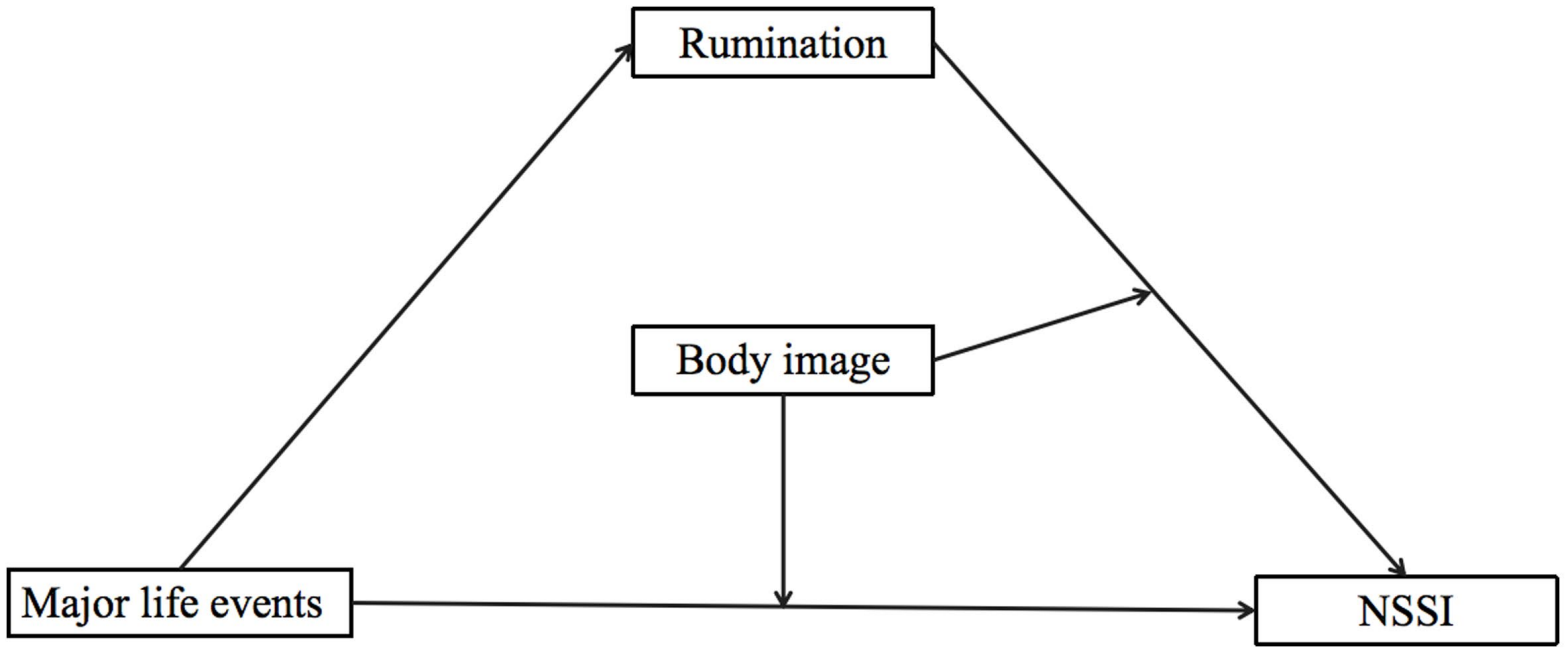
Hypothesis model.

In summary, the present study aims to further explore whether the relationship between major life events and NSSI is mediated by rumination. This examination is based on the emotional cascade theory of NSSI and Hooley and Franklin’s benefit and barrier theory. Additionally, this study aims to explore whether the relationship between rumination and NSSI and the relationship between major life events and NSSI are all moderated by body image. The hypothesized diagram of the model for this study is shown in Figure 1, and we propose the following three hypotheses:

*Hypothesis 1:* Major life events are positively related to NSSI.

*Hypothesis 2:* Rumination mediated the relationship between major life events and NSSI.

*Hypothesis 3:* Body image moderated the relationship between major life events and NSSI, as well as the relationship between rumination and NSSI.

## 2 Methods

### 2.1 Participants and procedure

A convenience sampling method was used to recruit 2,992 college students from a college located in southern China. All participants completed the questionnaires anonymously and received a detailed mental health report upon completion of the questionnaires. The questionnaires were distributed using the Questionnaire Star platform.^1^ Students can complete the questionnaires by scanning a QR code with their mobile devices or by accessing a link on their computers. Several measures were implemented to ensure the quality of the questionnaire. For example, for a fixed-option lie-detecting question, choose C for this question, answer time limited and so on. Blank options, answer time less than 8 min, and outliers were excluded from the final dataset. Ultimately, 2,717 valid questionnaires were collected, with an effective recovery rate of 90.81%. This study was approved by the Ethics Committee of the School of Psychology, South China Normal University, with the assigned ethical approval number SCNU-PSY-2022-217. All participants provided their informed consent by signing a consent form and were informed of their right to withdraw from the study at any time.

### 2.2 Measures

#### 2.2.1 Major life events

Major life events were measured using the Adolescent Self Rating Life Events Checklist compiled by Liu et al. (31) and subsequently revised by Li et al. (9). This checklist employed a six-point Likert-type scale ranging from 0 (did not occur) to 5 (did occur and there was heavy impact). A total of 16 items were included in the checklist, which assessed the stressors experienced by college students in various domains, including school, family, interpersonal, and personal factors. Examples of stressors included “conflicts or fights with classmates or close friends.” The total score was calculated by summing the responses to all items. Higher scores on the checklist indicated that the subjects suffered from more serious major life events. The scale had a good internal consistency with Cronbach’s alpha of 0.870.

#### 2.2.2 Non-suicidal self-injury

The non-suicidal self-injury was assessed using the Inventory of Statements about Self-harm (ISAS), a scale developed by Klonsky et al. (32) and translated by You et al. (33). This self-report inventory utilizes a six-point Likert-type scale to assess the severity of NSSI. It assesses the frequency of NSSI occurrences within the past year. The severity of NSSI among college students was assessed by inquiring about the frequency of engaging in 12 distinct methods of self-harm in the past year, e.g., intentionally injuring themselves with a fist, slap, or harder objects. In the given scale, where numerical values ranging from 0 to 5, the total score on the scale serves as an indicator of the severity of self-injurious behaviors, with higher scores representing more severe self-injury among college students. Cronbach’s alpha for the scale was 0.845.

#### 2.2.3 Rumination

Rumination was measured using the Ruminative Response Scale (RRS) developed by Nolen-Hoeksema et al. in 1991 (34) and further revised by Han et al. (35). The questionnaire consists of 22 items, with one example question being “I always think about a recent situation, wishing it had gone better.” The scale is assessed using a four-point Likert-type scale, ranging from 1 (never) to 4 (always), with the overall score being calculated. The higher score on the scale represents the more serious tendency of the rumination thinking of college students. The scale had a good internal consistency with Cronbach’s alpha of 0.974.

#### 2.2.4 Body image

Perceptions of body image of college students were measured using the Body Image State Scale (BISS) developed by Cash et al. (27) and translated and revised by Wang et al. (36) into Chinese version. The scale consists of six items, such as “how I feel about my body type at this moment” and “how I feel I look compared to the average person.” The scale is a nine-point Likert scale ranging from 1 (extremely dissatisfied) to 9 (extremely satisfied). The total score of the scale was calculated. Higher scores on the scale exhibited that the individuals have greater satisfaction with their body feelings and a more positive body image. The scale had a good internal consistency with Cronbach’s alpha of 0.973.

### 2.3 Covariates

This study incorporates demographic variables as covariates for analysis, which have been identified in previous research as possible predictors of the study outcomes (22), including gender, age, ethnicity, place of residence, subjective economic status, only child, history of mental illness, history of smoking, history of alcohol consumption, history of relationships, and somatic symptoms.

### 2.4 Statistical analysis

SPSS 26.0 was used for the initial analysis of the data and covariance test, and the data were tested for common method bias using Harman’s one-way test. The main variables were then tested for correlation using Spearman correlation analysis. The data were standardized for the main variables, and then the model was built and validated using Hayes’ PROCESS 4.0 plug-in, with Hypotheses 1 and 2 being tested by the mediated model (Model 4) and Hypothesis 3 being tested by the moderated mediation model (Model 15). While the bias-corrected nonparametric percentile Bootstrap methods was used to test the moderated mediation effect and estimate the 95% confidence interval with 5000 repetitions.

## 3 Results

### 3.1 Collinearity test

The variance inflation factor (VIF) of all predictive variables (1.078–1.353) was less than 3, and tolerance (0.739–0.928) was greater than 0.1, indicating that there is no serious multicollinearity in date (37).

### 3.2 Common method bias

To control the problem of common method bias, this study used the Harman one-way test for common method bias. The results showed that there were eight common factors were greater than 1. The unrotated first factor explained only 30.55% of the total variance and did not account for 40% of the total variance explained. This indicates that there is no significant common method bias in the data of this study.

### 3.3 Characteristics of the participants

As shown in Table 1, 2,717 college students were included in this study, 611 individuals (22.49%) were male, 1,050 individuals (38. 65%) resided in cities, and 367 individuals (13.51%) were only child. The prevalence of non-suicidal self-injury (NSSI) among college students was found to be 11.56%.

**Table 1:** Demographic characteristics of the sample.

Variable	*N* = 2,717	
Gender, n (%)		
Male	611	(22.49)
Female	2,106	(77.51)
Age, M (IQR)	19.81	(1.09)
Ethnicity, n (%)		
Han*	2,661	(97.94)
Minority	56	(2.06)
Place of residence, n (%)		
City	1,050	(38.65)
Rural	1,667	(61.35)
Economic status, n (%)		
Much better	41	(2.51)
Better	250	(9.20)
Similar	1,428	(52.56)
Worse	778	(28.63)
Much worse	220	(8.10)
Only child, n (%)		
Yes	367	(13.51)
No	2,350	(86.49)
History of mental illness, n (%)		
Yes	30	(1.10)
No	2,687	(98.90)
History of smoking, n (%)		
Never	2,537	(93.37)
Past	95	(3.50)
Present	85	(3.13)
History of alcohol consumption, n (%)		
Never	2,171	(79.90)
Past	180	(6.63)
Present	366	(13.47)
History of romantic relationships, n (%)		
Never	1,324	(48.73)
Previous	745	(27.42)
Present	648	(23.85)
Somatic symptoms, n (%)		
Yes	787	(28.97)
No	1930	(71.03)

*In China, the Chinese population is composed of 56 ethnic groups, known as Minzu, with the Han ethnic group (Han Zu) being the largest, accounting for 92% of the total population.

### 3.4 Descriptive analysis and correlation test of scale scores

Descriptive statistics and Spearman’s correlation analysis were performed for the main variables. As shown in Table 2, the results indicated that major life events were positively related to rumination (*r* = 0.52, *p* < 0.001) and NSSI (*r* = 0.24, *p* < 0.001), and rumination also showed a positive correlation with NSSI (*r* = 0.31, *p* < 0.001). Meanwhile, the results of the study showed that body image was negatively related to major life events (*r* = −0.22, *p* < 0.001), rumination (*r* = −0.29, *p* < 0.001), and NSSI (*r* = −0.18, *p* < 0.001).

**Table 2:** Descriptive statistics and correlation between the main variables (*N* = 2,717).

Variables	*M* ± *SD*	1	2	3	4
Major life events	7.48 ± 8.57	—			
Rumination	34.94 ± 13.12	0.52***	—		
Non-suicidal self-injury	0.73 ± 3.42	0.24***	0.31***	—	
Body Image	31.02 ± 10.22	−0.22***	−0.29***	−0.18***	—

^***^p < 0.001.

### 3.5 Mediation effect test

**Figure 2:**
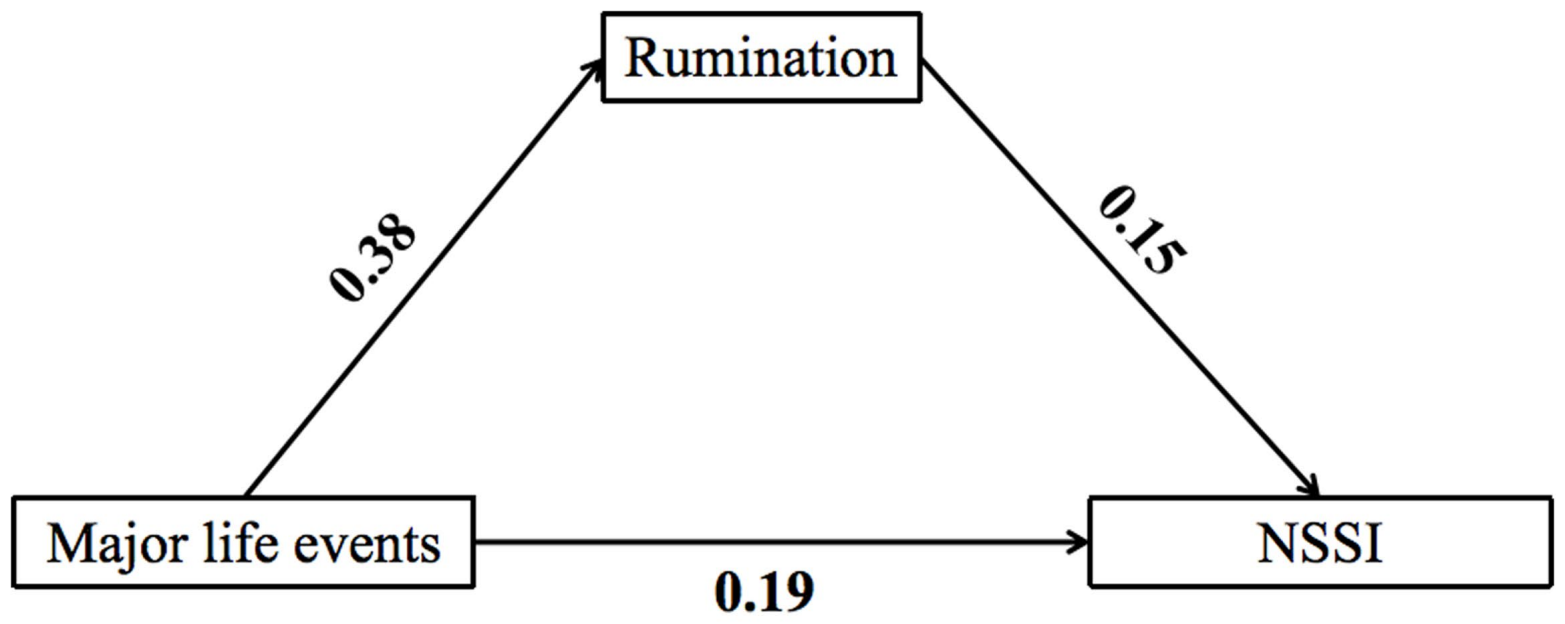
Mediation model (Model 4).

As shown in Figure 2 and Table 3, there was a positive correlation between major life events and NSSI (β = 0.218, *p* < 0.001). The inclusion of rumination as a mediating variable maintained the significant positive relationship between major life events and NSSI (*β* = 0.189, *p* < 0.001). In addition, there was a significant positive correlation between major life and rumination (*β* = 0.382, *p* < 0.001), and rumination was also positively related to NSSI (*β* = 0.146, *p* < 0.001). The findings indicated that major life events were not only directly related to NSSI but also indirectly related to NSSI through the mediating effect of rumination, which accounted for 33.03% (0.072/0.218) of the mediating effect.

**Table 3:** Total, direct, and indirect effects of major life events on NSSI.

	Effect	Boot SE	95% Boot LLCI	95% Boot ULCI
Direct effect	0.146	0.021	0.105	0.187
Indirect effect	0.072	0.012	0.051	0.096
Total effect	0.218	0.020	0.180	0.256

### 3.6 Moderated mediation effect test

**Figure 3:**
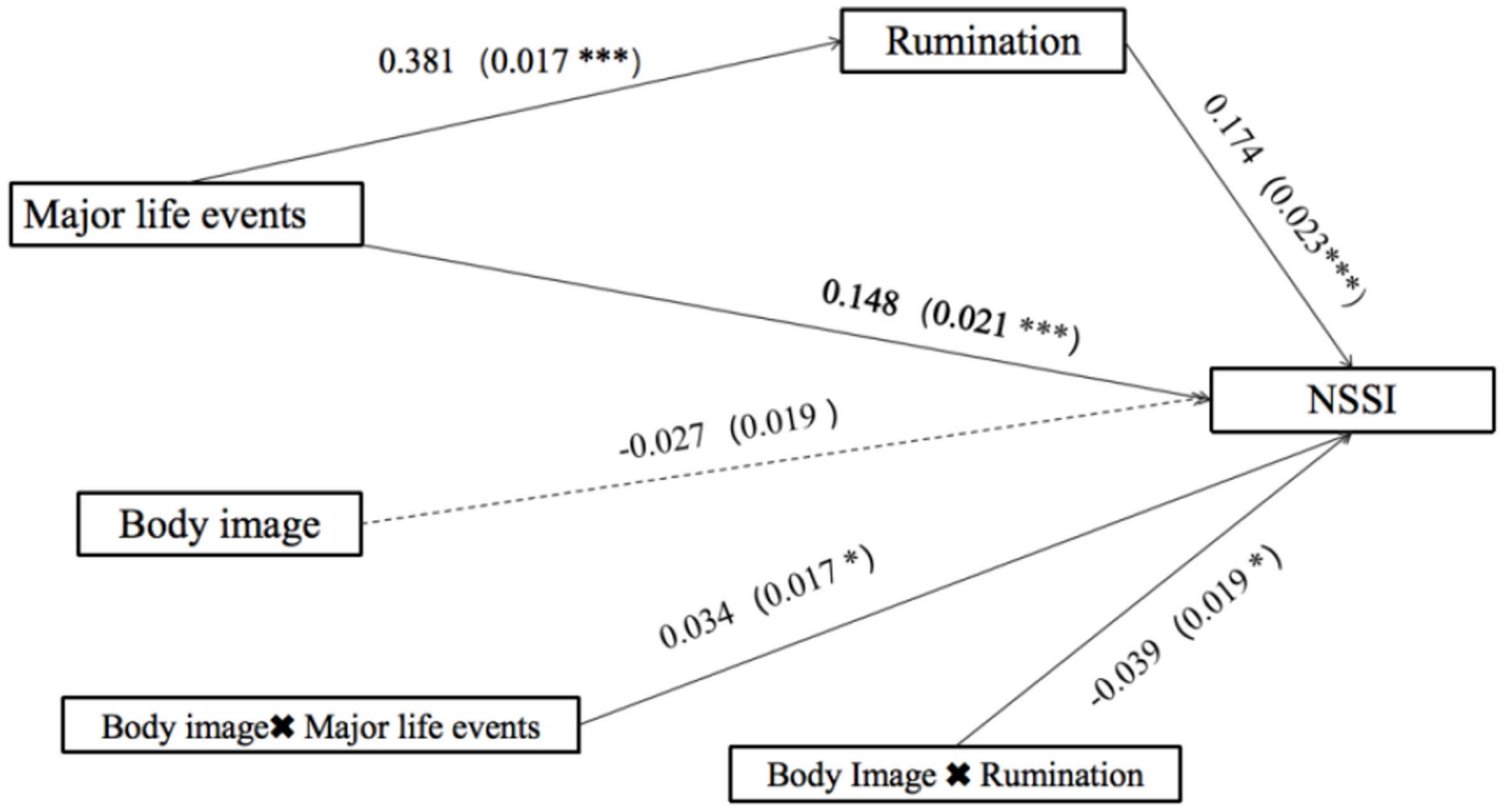
Moderated mediation model (Model 15).

Moderated mediation model tests were conducted using Model 15 of PROCESS 4.0, and the model plot is shown in Figure 3. The direct effect of major life events on NSSI was significantly moderated by body image (*β* = 0.034, 95% Cl: [>0.000, 0.068], *p* < 0.05). In addition, body image moderated the relationship between rumination and NSSI (*β* = −0.039, 95% Cl: [−0.076, −0.002], *p* < 0.05).

**Figure 4:**
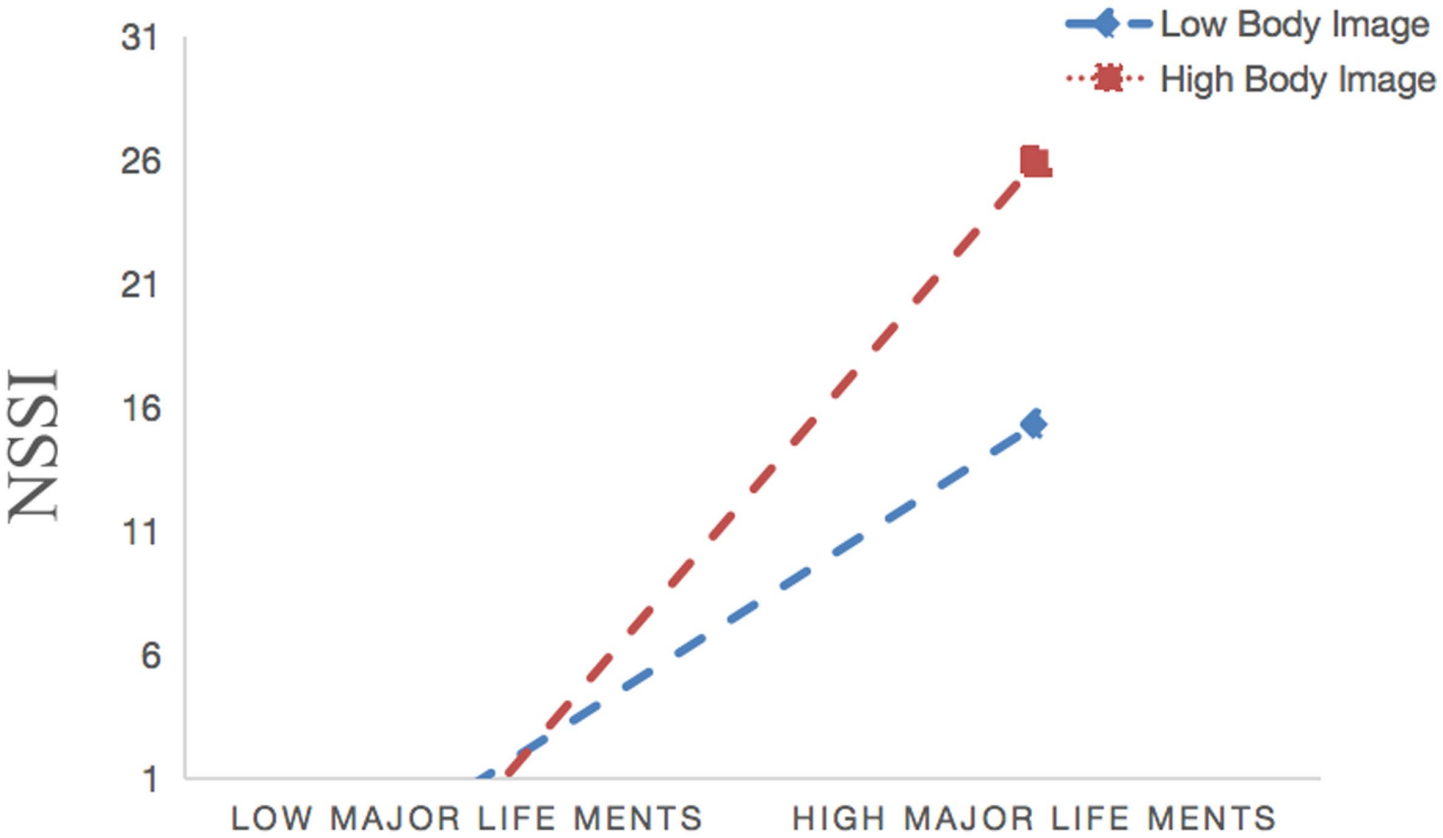
Interaction effect of body image and major life events on NSSI.

**Figure 5:**
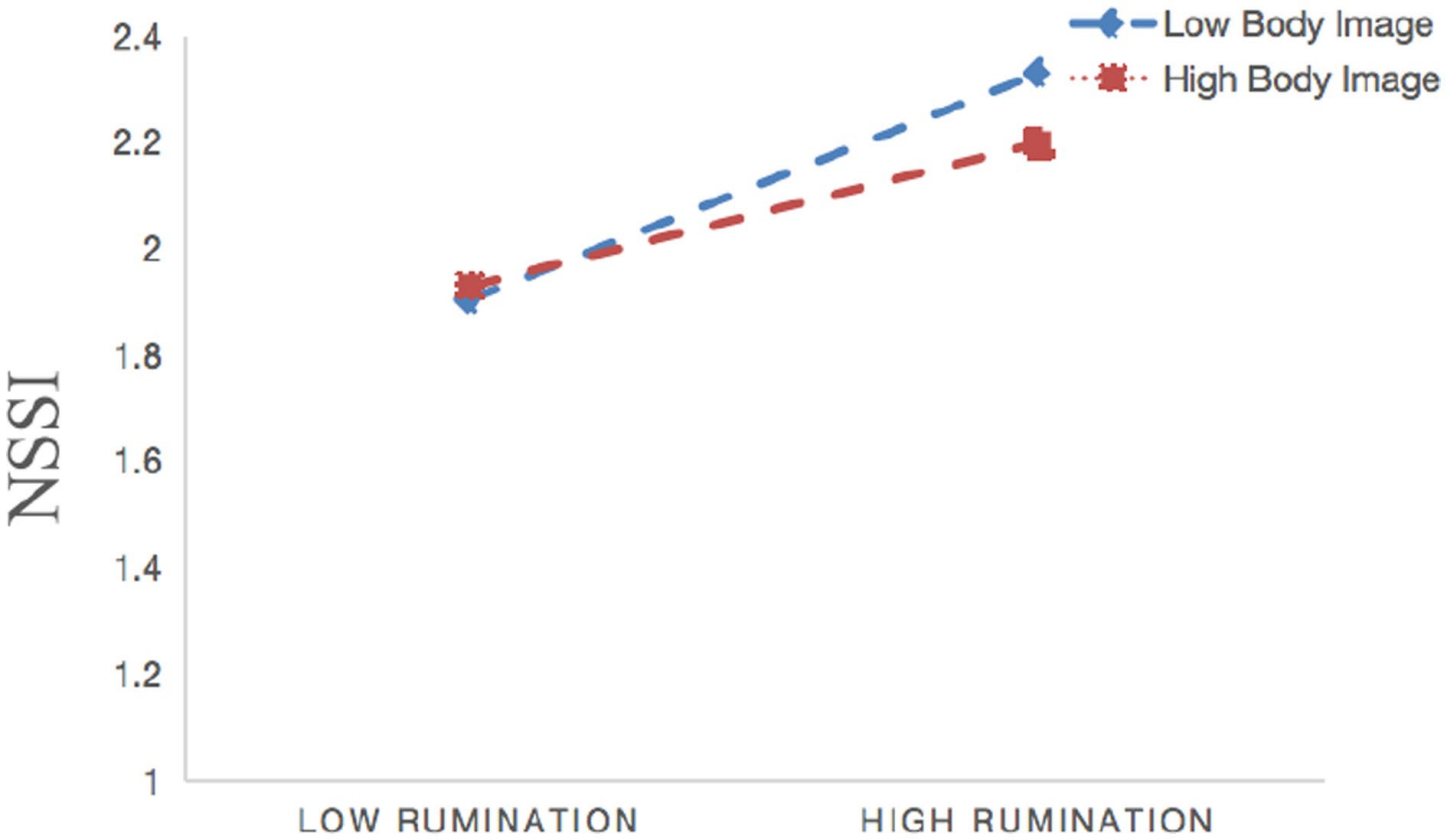
Interaction effect of body image and rumination on NSSI.

As shown in Figure 4, a simple slope analysis indicated that when individual’s satisfaction of their body image increased (on the basis of adding or subtracting one standard deviation), the correlation between major life events and the NSSI was strengthened (*β* = 0.125, *t* = 5.335, *p* < 0.001) vs. (*β* = 0.185, *t* = 6.387, *p* < 0.001). Whereas as shown in Figure 5, a simple slope analysis indicated that when individual’s level of satisfaction with the body image increased (on the basis of adding or subtracting one standard deviation), the correlation between rumination and NSSI was weakened (*β* = 0.201, *t* = 8.669, *p* < 0.001) vs. (*β* = 0.132, *t* = 3.850, *p* < 0.001).

## 4 Discussion

The present study revealed a prevalence rate of 11.56% for non-suicidal self-injury (NSSI) within the college student population. This finding matches closely with the results reported in previous research (6). One possible explanation for the high prevalence rate of NSSI among Chinese college students is that some students may be geographically separated from their families for the first time in their lives. This transition has the potential to increase their vulnerability to emotional dysregulation and pose challenges in accessing support from both familial and social networks (38, 39). According to Sawyer et al., college students, specifically those aged 18–22 years, can still be categorized as belonging to the adolescent developmental stage (1). Evidence suggests that this group continues to be vulnerable to the incidence of non-suicidal self-injury (1, 40). Hence, additional investigation is warranted to explore the potential risk factors and mechanisms of occurrence associated with this particular association.

The findings of the research additionally provide evidence in support of the hypothesis that major life events and rumination play a significant role in non-suicidal self-injury (Hypothesis 1). According to emotion appraisal theory, emotions arise from people’s interpretations and explanations of the environment in which they find themselves. According to Richard Lazarus, there are two main types of reactions to major life events: automatic, unconscious, and rapidly activated emotional responses, and conscious emotional responses related to how to cope, such as rumination (20). Maladjustment in individuals can occur when they experience negative emotion and engage in negative thinking (41, 42). They tend to release those negative emotions by choosing NSSI, which is relatively easy to obtain and does not require any tools (43). This finding is consistent with Nock’s integrative model of NSSI (44). Individuals who are exposed to higher frequency or more severe life events tend to exhibit higher levels of non-suicidal self-injury (NSSI) (45). Additionally, engaging in rumination, which involves persistent negative emotions, makes individuals more vulnerable to becoming trapped in negative emotional states (46). Consequently, this susceptibility to prolonged rumination can contribute to the escalation of NSSI behaviors, resulting in greater severity (19, 47).

The present study provides additional evidence for Hypothesis 2, which is derived from the experiential avoidance theory and emotional cascade theory of non-suicidal self-injury (NSSI). Specifically, it posits that persons who experience major life events are more prone to experiencing negative feelings. Which are stacked one on top of the other and constantly reverberate in the individual’s mind, making it easy for them to constantly ruminate and get stuck in an emotional rut (24, 48) and thus unable to get out of it, and in order to better dissipate this emotion. Individuals frequently engage in self-harming behaviors as a way of easing their psychological distress, leading to persistent NSSI behavior (49). The findings highlight the significant mediating role of rumination in the relationship between major life events and NSSI. Inter-individual differences are observed within various groups, and certain individuals may engage in NSSI, immediately following a major life event as a way to reduce the negative emotions associated with such event (50). Nevertheless, it is worth noting that certain individuals may not exhibit immediate reactions to major events. Instead, they may engage in a process of contemplation and recollection, often facilitated by rumination. In cases where negative emotions persist and intensify to a certain degree, there is an increased likelihood of the individual developing non-suicidal self-injury behaviors (25, 51).

The results also showed that body image significantly moderated the relationship between rumination and NSSI. Specifically, individuals with a positive body image demonstrated a weaker association between rumination and NSSI compared with those with a negative body image. A stronger relationship is found between rumination and non-suicidal self-injury among those who have poor body image perception. This result is consistent with previous research, implying that body image may serve as a protective factor against NSSI (52). Individuals with high body intention are less likely to engage in NSSI (53). Surprisingly, it is found that body image significantly moderated the relationship between major life events and NSSI. Major life events have a stronger relationship with NSSI in people who have a positive body image. Conversely, individuals with a negative body image exhibit a weaker relationship between major life events and NSSI. This result could be related to the link between individuals with a positive body image and have a higher self-perception. Individuals who have a strong sense of high self-esteem may experience a greater sense of discrepancy when confronted with a major life event that proves difficult for them to accept (54–56). Consequently, this amplification of emotional difficulties may contribute to an increased likelihood of engaging in non-suicidal self-injury (57).

Drawing from these findings, we suggest that the perception of body image cannot be studied from a single latitude. One potential perspective is that individuals who have a positive body image may be less likely to engage in self-harm behaviors (58). On the contrary, it is important to acknowledge the potential negative consequences of having high levels of body intentions. Individuals with attractive appearances frequently garner admiration and favor from others. However, this can also pose difficulty for them in managing negative emotions and severe major life events. Consequently, visually attractive individuals are more likely to engage in non-suicidal self-injury (53, 59). It is suggested that there may exist a non-linear association between body image, self-esteem, and self-concept with non-suicidal self-injury (NSSI), perhaps manifesting as an inverted “U”-shaped relationship. Additional research is needed. The current research also further provides partial support for Hooley and Franklin’s benefits and barriers model of NSSI (26).

While this study enriches our understanding of NSSI to a certain degree and produces intriguing findings, there are some limitations that need to be discussed. First, the current study employed a cross-sectional design. Although it provides some insight into the association among the four variables, it is important to note that it is not possible to establish causal conclusions. Therefore, it is recommended that future studies research endeavors undertake more longitudinal studies. Second, this study employed self-report questionnaires. Although the questionnaires were collected anonymously, the participants may have been affected by the stigmatization of the NSSI and the social expectancy effect. Future studies should use as many evaluation methods as possible. This study proposes the possibility of a non-linear association between body image and non-suicidal self-injury (NSSI), highlighting the need for additional investigation in this area.

In summary, the current research expands upon existing research by investigating the underlying mechanism linking major life events and NSSI among college students. The current research is the first research in establishing a relationship between major life events and non-suicidal self-injury (NSSI), with rumination identified as a mediating factor between the two and the moderating role of bodily image in different pathways. It is imperative for parents, school workers, and clinicians to not only acknowledge the major life occurrences that college students encounter but also assist them in acquiring additional strategies for emotional regulation and mitigating the occurrence of emotional rumination. Parents, teachers, and healthcare providers should provide more services to help students mitigate negative rumination, such as rumination-based cognitive behavioral therapy (60, 61) and mindfulness therapy (62).

In the context of teenagers experiencing emotional difficulties, enhancing their body image can potentially lead to an increased appreciation of their physical appearance, hence perhaps reducing the occurrence of non-suicidal self-injury (NSSI) behaviors. On the other hand, attention should also be paid to the ability of college students to withstand setbacks. Higher self-evaluation may also produce undesirable results, such as it is more difficult to accept that life has become bad and make people more difficult to withstand major events in life. We hypothesize that there may exist a non-linear association between body image, self-esteem, and self-concept with non-suicidal self-injury (NSSI), perhaps manifesting as an inverted “U”-shaped relationship, but this hypothesis requires further research.

## Data availability statement

The datasets generated and/or analysed during the current study are available from the corresponding author on reasonable request.

## Ethics statement

The studies involving humans were approved by the Ethics Committee of the School of Psychology, South China Normal University. The studies were conducted in accordance with the local legislation and institutional requirements. The participants provided their written informed consent to participate in this study.

## Author contributions

HZ: Conceptualization, Methodology, Project administration, Writing – review & editing. WZ: Conceptualization, Methodology, Project administration, Writing – review & editing. YL: Conceptualization, Methodology, Writing – review & editing. WW: Conceptualization, Methodology, Writing – review & editing. JW: Conceptualization, Methodology, Writing – review & editing. QQ: Conceptualization, Methodology, Writing – original draft. GY: Conceptualization, Methodology, Writing – original draft. CZ: Formal analysis, Writing – review & editing. XK: Formal analysis, Writing – review & editing.

## Footnotes

^1^ www.wjx.cn
